# A Critical Interpretive Synthesis of the Role of Arecoline in Oral Carcinogenesis: Is the Local Cholinergic Axis a Missing Link in Disease Pathophysiology?

**DOI:** 10.3390/ph16121684

**Published:** 2023-12-04

**Authors:** Hakan Gocol, Jin Han Zeng, Sara Chang, Buo Yu Koh, Hoang Nguyen, Nicola Cirillo

**Affiliations:** Melbourne Dental School, The University of Melbourne, Carlton, VIC 3053, Australiabuoyuk@student.unimelb.edu.au (B.Y.K.);

**Keywords:** arecoline, nicotine, acetylcholine receptors, oral submucous fibrosis, oral cancer, mouth neoplasms

## Abstract

Arecoline is the primary active carcinogen found in areca nut and has been implicated in the pathogenesis of oral squamous cell carcinoma (OSCC) and oral submucous fibrosis (OSF). For this study, we conducted a stepwise review process by combining iterative scoping reviews with a post hoc search, with the aim of identifying the specific mechanisms by which arecoline initiates and promotes oral carcinogenesis. Our initial search allowed us to define the current trends and patterns in the pathophysiology of arecoline-induced OSF and OSCC, which include the induction of cell proliferation, facilitation of invasion, adhesion, and migration, increased collagen deposition and fibrosis, imbalance in immune and inflammatory mechanisms, and genotoxicity. Key molecular pathways comprise the activation of NOTCH1, MYC, PRDX2, WNT, CYR61, EGFR/Pl3K, DDR1 signaling, and cytokine upregulation. Despite providing a comprehensive overview of potential pathogenic mechanisms of OSF, the involvement of molecules functioning as areca alkaloid receptors, namely, the muscarinic and nicotinic acetylcholine receptors (AChRs), was not elucidated with this approach. Accordingly, our search strategy was refined to reflect these evidence gaps. The results of the second round of reviews with the post hoc search highlighted that arecoline binds preferentially to muscarinic AChRs, which have been implicated in cancer. Consistently, AChRs activate the signaling pathways that partially overlap with those described in the context of arecoline-induced carcinogenesis. In summary, we used a theory-driven interpretive review methodology to inform, extend, and supplement the conventional systematic literature assessment workflow. On the one hand, the results of this critical interpretive synthesis highlighted the prevailing trends and enabled the consolidation of data pertaining to the molecular mechanisms involved in arecoline-induced carcinogenesis, and, on the other, brought up knowledge gaps related to the role of the local cholinergic axis in oral carcinogenesis, thus suggesting areas for further investigation.

## 1. Introduction

Arecoline, an active carcinogenic alkaloid found in areca nut, has been recognized as an important factor in the pathogenesis of premalignant and malignant oral disorders [1], specifically, oral submucous fibrosis (OSF) and oral squamous cell carcinoma (OSCC). However, the precise molecular mechanisms underlying arecoline-induced OSF and OSCC development remain unclear [2,3].

OSF is characterized by progressive fibrosis and inflammation of the submucosal tissues [4]. It is a potentially malignant disorder that is associated with an increased risk of OSCC, a malignant neoplasm originating from the stratified squamous epithelium of the oral mucosa [5]. OSF progression to OSCC takes place in approximately 7–14% of patients [6]. OSCC is one of the most frequently reported malignancies in the world, especially in Taiwan and India, accounting for approximately 90% of all oral cavity cancers [7].

Previous research has identified key biological changes underlying arecoline-induced oral carcinogenesis, including abnormal cell proliferation [8], the dysregulation of cell cycle control [9], invasion and metastasis [10], fibrotic alterations [11], altered inflammatory and immune responses [12], and epigenetic modifications [13]. However, the specific signatures associated with arecoline-induced oral carcinogenesis and their roles in disease pathogenesis are widely heterogeneous.

Understanding the mechanisms that are specifically related to the effect of arecoline in the oral mucosa is particularly challenging. In humans, dissecting the arecoline-specific pathways leading to carcinogenesis is virtually impossible as areca nut contains several potentially detrimental alkaloids [14]. In addition, areca nut is often consumed together with betel leaves and other ingredients in a mixture—the so-called betel quid (BQ). Different BQs have diverse chemical compositions and the interaction of different constituents confers a distinct disease-inducing capacity to the quids [15]. Hence, conventional systematic review methodology may be inappropriate in tackling this question.

Critical interpretive synthesis draws on the traditions of qualitative research inquiry and systematic review methodology and can be used to synthesize both qualitative and quantitative forms of evidence [16]. This methodology is explicitly oriented toward theory-building and proposes an iterative and dynamic approach to question formulation, searching, and the selection of materials for inclusion in reviews. For the systematic assessment of the existing literature, a scoping review was deemed to be the most suitable approach as it comprehensively examines the existing literature, mapping key concepts, evidence sources, and knowledge gaps. This approach provides an overview, identifies the research trends, and highlights areas for further investigation [17].

Our study aims to investigate the mechanisms through which arecoline promotes the progression of OSCC. Understanding the molecular pathways and diagnostic markers associated with arecoline-induced oral carcinogenesis is paramount for the early detection, prevention, and, potentially, treatment of oral cancer.

## 2. Results

### 2.1. Initial Scoping Review

**Figure 1:**
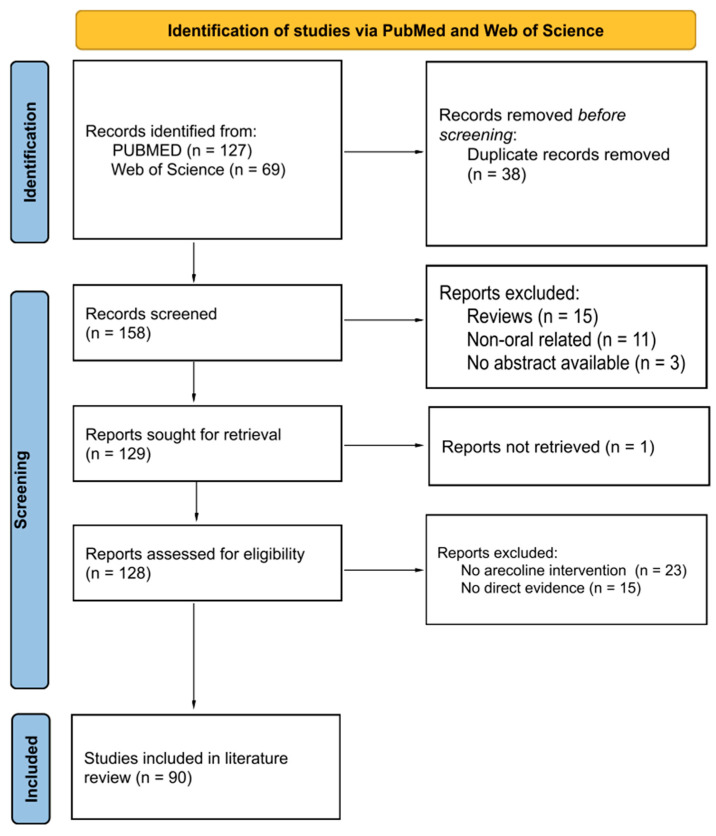
PRISMA flow diagram demonstrating the selection process for the initial scoping review. PRISMA: preferred reporting items for systematic reviews and meta-analysis.

The first round of this review assessing the mechanisms of arecoline-associated carcinogenesis identified 196 records, with 90 eligible articles included in the qualitative synthesis (Figure 1). Of these 90 studies, there were 14 in vivo (Table 1) and 86 in vitro investigations (Supplementary Table S1), with multiple studies containing both in vitro and in vivo components. Most of the in vitro studies reported on the molecular markers involved in arecoline-induced oral carcinogenesis (Supplementary Table S1). There were 32 studies that used fibroblasts, 23 studies that used keratinocytes, and 1 that involved endothelial cells.

#### 2.1.1. Evidence of Increased Cell Growth and Proliferation

In vivo studies on murine models exposed to arecoline revealed the presence of squamous cell hyperplasia in the excised samples [8,18,19,20]. The introduction of arecoline-stimulated OSCC cell lines resulted in tumor growth [21]. Furthermore, certain studies explored the gene expressions associated with cellular proliferation and noted that arecoline promoted the gene expression of notch receptor 1 (NOTCH1), protein tyrosine kinase 6 (PTK6), and discoidin domain receptor tyrosine kinase 1 (DDR1) [8,10,12,22] while downregulating the tumor suppressor gene, retinoic acid receptor beta (RARB) [23].

Numerous in vitro studies have demonstrated that arecoline exposure leads to increased cell proliferation in various oral cell lines, including OSCC cells, oral keratinocytes, and gingival fibroblasts [8,10,24,25,26,27]. The levels of numerous biomarkers of cell proliferation, such as proliferating cell nuclear antigen (PCNA) and antigen kiel 67 (Ki67), were found to be elevated with arecoline treatment [8,24]. Studies that investigated gene expression found that arecoline triggers the upregulation of a wide range of genes and signaling pathways, including NOTCH1, MYC proto-oncogene (MYC), peroxiredoxin 2 (PRDX2), Wnt pathway (WNT), cysteine-rich angiogenic inducer 61 (CYR61), epidermal growth factor receptor/phosphoinositide 3-kinases (EGFR/Pl3k), and discoidin domain receptor 1 (DDR1) [8,12,22,28,29,30,31]. In contrast, the expression of tumor suppressor genes such as alcohol dehydrogenase, iron-containing 1 (ADHFE1), aldehyde dehydrogenase 1 family member A2 (ALDH1A2), dual specificity phosphatase 4 (DUSP4), and tumor protein p53 (TP53) was found to be downregulated upon exposure to arecoline [32,33,34]. In one study, arecoline was shown to decrease cell survival and proliferation in a dose-dependent manner [35].

#### 2.1.2. Apoptosis/Cell Cycle Arrest

Caspase 8 (CASP8) was upregulated in mice models challenged with arecoline N-oxide (ANO) [20]. In vitro, arecoline induction was found to result in keratinocyte cell proliferation and inhibit apoptosis via PRDX2 gene overexpression [29], the elevation of CASP8 protein [20], the activation of mitogen-activated protein kinase 1/extracellular-signal-regulated kinase pathway (MEK1/ERK pathway) [36], and through triggering the ATM-dependent pathway, inducing arrest at mitosis [37,38]. There were observed increases in transcription factor Jun (c-jun) mRNA levels with fos proto-oncogene (c-fos) pathway activation, affecting cell cycle progression [36], but it was reported elsewhere that this did not induce c-fos mRNA expression [9].

Exposure of epithelial cells to arecoline was found to suppress viability and promote apoptosis and atrophy in a dose-dependent manner [26,39]. Arecoline inhibits epithelial cell proliferation and affects cell morphology, including cell cycle arrest in the G1/S phase and survival in a dose-dependent manner [35,40,41].

For SAS cancer cells, arecoline leads to cell death, apoptosis, and cell cycle arrest by stimulating checkpoint kinase 1 (Chk1) and checkpoint kinase 2 (Chk2) phosphorylation [42].

In fibroblasts, arecoline inhibited the expression and function of tumor protein p53 (p53) and its downstream molecules [27,34,38], as well as down-regulated cyclin-dependent kinase inhibitor 1 (p21) and cyclin-dependent kinase inhibitor 1B (p27) [43], increased carbonic anhydrase IX (CAIX) expression [44], and led to cell-cycle exit [45]. Arecoline was also found to inhibit the tissue inhibitor of metallopeptidase 1 (TIMP-1) and tissue inhibitor of metalloproteinase 2 (TIMP-2) in fibroblasts in one in vitro study [42]; however, TIMP-1 was elevated in two other studies [46,47].

**Table 1:** Summary of the in vivo studies included in the scoping review *.

Author, Year	Population/Model/Intervention	Outcomes/Diagnostic Markers Measured	Results Observed
Ren et al., 2021 [48]	BALB/c nude mice, injected with OSCC (CAL33 cell line) with or without arecoline	Tumor size and cervical LN metastasis; SAA1 levels (cytokine)	No significant difference in tumor size; 50% increase in mice with cervical LN metastasis; elevated SAA1 levels
Nithiyanantham et al., 2021 [12]	NOD/SCID mice were challenged with ANO	NOTCH1 and cytokines (IL17a, IL1B)	ANO led to elevated NOTCH1, Il-1α, Il-1β
Kuo et al., 2019 [8]	C57BL/6 mice were challenged with either arecoline or ANO	Gross and histopathological changes; NOTCH1, FAT1, HES1	Arecoline/ANO induced squamous hyperplasia, leukoplakia, collagen deposition, and elevation of NOTCH1, FAT1, and HES1
Li et al., 2021 [21]	BALB/c nude mice, injected with OSCC (CAL27 cell line) with different conditions: FTO overexpression (induced by arecoline) and knockdown	Tumor growth; cytokines (TNF-α, IFN-γ, TGF-β, IL-10, IL-17)	Arecoline-induced FTO overexpression significantly increased tumor growth, increased TGF-β, IL10, and IL17, and reduced TNF-α and IFN-γ
Hsieh et al., 2022 [10]	C57BL/6J mice were challenged with 4-nitroquinoline 1-oxide (4-NQO) and arecoline	PTK6 methylation level, PTK6	4-NQO and arecoline reduced the methylation of PTK6 and elevated PTK6
Huang et al., 2020 [18]	C57BL/6J mice were challenged with 4-NQO and arecoline	Gross and histopathological changes	4-NQO and arecoline induced gross white lesions with squamous hyperplasia and dysplasia
Kuo et al., 2015 [19]	NOD SCID mice were challenged with ANO	Histopathological changes	ANO induced epithelial squamous hyperplasia and increased collagen deposition
Hu et al., 2022 [49]	C57/BL6 mice were challenged with arecoline	Histopathological changes; DEC1, FAK, and Akt levels	Arecoline led to OSF and fibrotic alteration and induced the elevation of DEC1/FAK/Akt
Chang et al., 2017 [20]	NOD/SCID mice and 3 C57BL/6JNarl mice were challenged with ANO	CASP8	ANO induced upregulation of CASP8 in both NOD-SCID and C57BL/6 mice; ANO induced upregulation of PCNA and Ki67 proteins in the sublingual squamous hyperplastic lesion
Chang et al., 2010 [50]	C57BL/6JNarl mice were challenged with arecoline, 4-NQO, or both arecoline and 4-NQO	aB-crystallin and Hsp27	aB-crystallin and Hsp27 were upregulated in murine oral tumors
Chen et al., 2016 [51]	Tg mouse lines in C57BL/6 were challenged with 4-NQO and arecoline	Presence of oral tumors	4-NQO successfully induced tumors on the tongue surface, in the esophagus, and occasionally on the palate or buccal mucosa
Zheng et al., 2018 [34]	Nude mice were injected with OSCC cell (SCC-9) followed by administration of arecoline	Tumor growth, Ki67, LSD1, E-cadherin, N-cadherin, and vimentin	Arecoline increased tumor growth and Ki67 expression. Arecoline decreased the expression of LSD1 and E-cadherin but increased the expression of N-cadherin and vimentin
Chiang et al., 2016 [52]	C57BL/6 mice at 6 weeks of age were challenged with 4-NQO and arecoline	Krt17	Krt17 was significantly upregulated in hyperplastic and carcinoma tissues
Lai et al., 2014 [23]	C57BL/6JNarl mice were challenged with 4-NQO, arecoline, or both	Retinoic acid receptor ß (RARB) promoter region	RARB promoter hypermethylation and loss of expression involved in areca-associated cancer

* See Abbreviations part for abbreviations and acronyms.

#### 2.1.3. Promoting Invasion (Migration/Epithelial-to-Mesenchymal Transition (EMT)/Adhesion/Invasion)

Growth and invasion were promoted through the arecoline-induced upregulation of the *NOTCH1* gene in mouse models [8,12]. EMT was promoted, activating invasion, and resulting in elevated PTK6 expression with E-cadherin (E-cad) suppression [10], which is deleted in esophageal cancer 1 (DEC1) upregulation, leading to focal adhesion kinase/serine/threonine kinase (FAK/AKT) [49], and through the upregulation of keratin 17 (Krt17) [52] in murine models.

In in vitro experiments, arecoline has been found to promote EMT in oral epithelial cells through DEC1 upregulation activating FAK/AKT downstream [49], the upregulation of proteasome activator complex subunit 3 (PA28γ), and the phosphorylation of MEK-1 [53] and was found to promote the expression of EMT-related genes [27,34]. Arecoline resulted in a dose- and time-dependent increase in zinc finger protein 1 (*SNAI1*) expression in human oral keratinocytes (HOKs) and OECM-1 [54]. The long-term exposure of buccal mucosal fibroblasts (BMFs) resulted in the dose-dependent upregulation of transcription factor zinc-finger E box-binding homeobox 1 (*ZEB1*) and the upregulation of insulin-like growth factor receptor 1 (IGF-R1) [55,56].

In terms of cellular adhesion, arecoline has been found to upregulate αvβ6 integrin expression in oral keratinocytes, modulated by the M4 muscarinic receptor in OKF6/TERT-1 cells [57]. Arecoline led to the increased attachment of U397 mononuclear cells to EAhy926 cells [58].

Arecoline upregulated COX-2 expression, affecting OSF cellular migration [59], leading to the dose-dependent elevation of the S100 calcium-binding protein A4 (*S100A4*) expression in oral epithelial (OE) cells [60,61], upregulation of alpha v beta 6 integrin (αvβ6) [57], elevation of TWIST [62], and upregulation of Lin-28 homolog B (Lin28B) expression [25]. A low dose of arecoline reduced the promoter methylation of protein tyrosine phosphatase receptor type M (*PTPRM*) and forkhead box D3 (FOXD3) in BMFs [2].

#### 2.1.4. Fibrotic Alteration/Impaired Wound Healing

Arecoline challenge resulted in squamous cell hyperplasia, increased collagen deposition and fibrotic alteration, and also increased cervical lymph node (LN) metastasis in mice [8,18,19,48,49].

Arecoline-treated BMFs resulted in a dose-dependent increase in SLUG protein, leading to the increased expression of type I collagen [11]. Collagen production was increased through heat shock protein (HSP) 47 upregulation and altered matrix metallopeptidase 1, 2, and 9 (MMP-1, MMP-2, and MMP-9) expression [42,45,47,63,64]. Extracellular matrix (ECM) synthesis and secretion was increased through the upregulation of *S100A4* gene expression [61]. Plasminogen activator inhibitor-1 (PAI-1) expression was dose-dependently elevated in arecoline-treated BMFs [65] but the increased expression of α-SMA (alpha smooth muscle actin) [52] was observed in one study, while decreased expression was seen in another [66].

Arecoline-treated BMFs resulted in collagen contraction inhibition, due to the suppression of the TWIST protein [62], and collagen phagocytosis was inhibited in a dose-dependent manner [67]; however, another study found that arecoline-induced OSF contraction through the induction of phospholipase C/inositol triphosphate 3/calcium/calmodulin (PLC/IP3/Ca^2^⁺/calmodulin), Ras homologous protein family (Rho) signaling pathways, and actin filament polymerization [68].

#### 2.1.5. Immune Responses and ROS/Antioxidant Activity

One in vivo study conducted on murine models observed increased antioxidant activity in heat shock protein 27 (HSP27) when the mice were exposed to arecoline [50].

Three in vivo studies investigated the effects of arecoline on different cytokines. Elevated levels of transforming growth factor-β (TGF-β) and interleukins (IL-1α, IL-1β, IL-10, and IL-17) were observed [12,21,48]. Tumor necrosis factor alpha (TNF-α) and interferon gamma (IFN-γ) were reduced in one of these studies [21].

Six in vitro studies observed ROS production in cells challenged with arecoline or its metabolites, including ANO, arecaidine, and arecoline N-oxide mercapturic acid (NOM) [12,26,54,68,69]. Antioxidant activities were shown to be reduced in some studies [12,69,70,71] but were increased in other studies [72,73,74].

Multiple in vitro studies have indicated the elevated expression of several inflammatory cytokines, including TGF-β, interleukins (IL-1α, IL-1β, IL-17α), serum amyloid A1 (*SAA1*), prostaglandin E2 (PGE2), and TNF-α [8,12,19,45,48,57,75,76,77,78]. However, one study observed a reduction in IL-6 and minimal changes in TNF-α [77]. One study mentioned the upregulation of programmed death-ligand 1 (PD-L1) in OSCC cells that were exposed to arecoline [28].

#### 2.1.6. Genotoxicity and Epigenetics

The induction of DNA damage and the alteration of repair mechanisms are widely regarded as the central mechanisms responsible for arecoline-induced carcinogenesis. In vitro, arecoline stimulated an increase in O6-methyl-guanine-DNA methyltransferase (MGMT) expression in HOKs and an increase in the phosphorylation of H2AX variant histone (γH2AX) [19,24,45], as well as the induced markers of irreparable DNA double-stranded breaks in normal human oral fibroblasts and p53-binding protein 1 (53BP1) [45]. Low doses of arecoline induced elevated cell proliferation and DNA repair [24]; however, long-term and high-dose exposure reduced DNA repair [69]. Arecoline also resulted in the reduced expression of sirtuin 1 (SIRT1) mRNA [79]. In contrast, a study found that arecoline is cytotoxic, while no genotoxicity was found in human buccal fibroblasts affecting DNA [80].

In terms of epigenetic regulation, arecoline upregulated microRNA (miRNA) miR-211 expression in OSCC cell cultures [51]. miR-211-promoted OSCC was shown to repress gene transcription factor 12 (*TCF12*) and peroxiredoxin-like 2A (FAM213A) [51]. Arecoline exposure to cultured cells such as HOKs and OSCC cell lines led to a reduction in miR-1455 [13], miR30a, miR379, miR-203, miR-22, miR-200b, miR329, and miR410 and an increase in miR-23a, miR-886-3p, and miR-10b [13,27,31,32,42,76,79,81,82,83]. miR-23a overexpression was found to be associated with reduced double-stranded break repair [82], while miR-200 was shown to be involved in arecoline-related myofibroblast activities in BMFs and fBMFs [42]. Arecoline inhibits miR-22, resulting in increased OSM levels and reduced function in OSCC cells [76]. Both miR329 and miR410 led to the promotion of tumor proliferation and invasion in oral carcinogenesis in cultured HOKs and OSCC cell lines [31].

### 2.2. Second Round of the Scoping Review, with a Post Hoc Search

The second scoping review focusing on the role of acetylcholine receptors in arecoline-induced carcinogenesis returned 16 results (PubMed, *n* = 6; Web of Science, *n* = 10). Following deduplication and screening, only one study was eligible for inclusion [57]. The post hoc manual search, including a proximity search of the studies identified in the second scoping review, offered mechanistic insights into the pathways activated by arecoline and allowed us to generate a framework that fits a model of receptor-mediated arecoline-induced oral carcinogenesis.

#### 2.2.1. Arecoline-Mediated Acetylcholine Receptor Signaling in Oral Carcinogenesis

**Figure 2:**
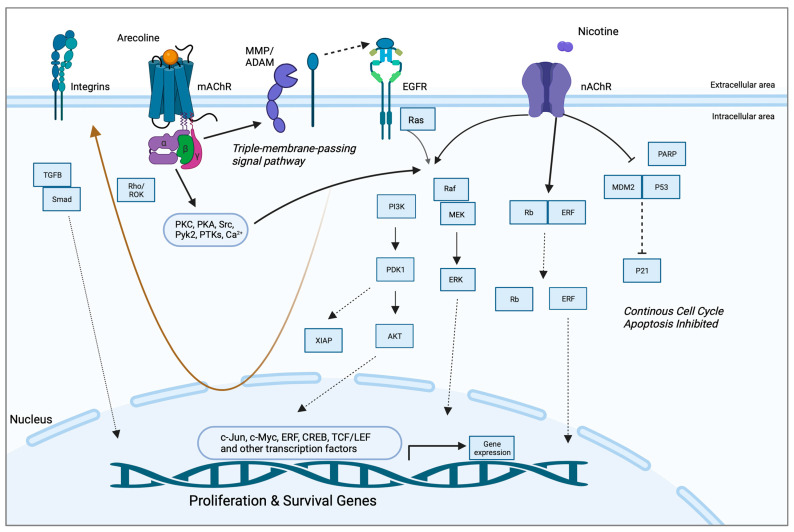
Putative pathways involving the receptor-mediated signaling of arecoline. Arecoline binds preferentially to muscarinic acetylcholine receptors (mAChR) but can also serve as a partial agonist of nicotinic receptors (nAChR). Two key molecular pathways involve EGFR and integrins. mAChR activates EGFR signaling via a so-called “triple-membrane-passing” pathway, whereby metalloproteinases cleave and activate EGF-like ligands, which, in turn, bind to EGFR and trigger downstream kinase signaling, including the Ras/Raf/MEK/ERK pathway. MAPK signaling can also be activated via canonical second messenger-mediated signals, as well as via nAChR. The two receptors also work synergistically to promote survival and inhibit apoptosis via PI3K/Akt and p21, respectively. Together, these pathways promote the expression of proliferation and survival genes, as well as migration/invasion and fibrosis/senescence via integrins and TGF-beta signaling, respectively (brown arrow). See Abbreviations part for the abbreviations and acronyms.

A study by Gareth Thomas’ group demonstrated that arecoline upregulated keratinocyte αvβ6 expression, a process modulated through the M(4) muscarinic acetylcholine receptor [57]. Arecoline-dependent αvβ6 upregulation promoted keratinocyte migration and induced invasion, raising the possibility that this mechanism may support malignant transformation. In another study, long-term nicotine-derived nitrosamine ketone (NNK) and arecoline exposure resulted in an increase in cancer stem cell properties, anti-apoptotic pathways, and a resistance to cisplatin in head and neck squamous cell carcinoma (HNSCC) cells in vitro [84]. The EGFR protein was pivotal in inducing tumor promotion and in impeding apoptosis in cancer cells by inducing phosphorylated AKT serine/threonine kinase 1 (pAKT) and nuclear factor kappa B (NFκB). While the authors pointed out that both NNK and arecoline exert agonist activity with the alpha-7-nicotinic acetylcholine receptor (α7-nAChR), the study did not directly investigate the role of nAChR in mediating the effects reported and, hence, was not included in the qualitative synthesis. Both studies, however, point to the possibility that arecoline promotes carcinogenesis via receptor-mediated mechanisms, an aspect that has not been captured in the available literature. The putative signaling pathways are depicted in Figure 2.

#### 2.2.2. The Effects of Arecoline in the Oral Mucosa Could Be Mediated by the Local Cholinergic Axis

Despite a substantial body of evidence demonstrating the pro-carcinogenic effects of arecoline, both in vivo and in vitro, our iterative scoping reviews failed to shed light on the receptor-mediated signaling that is probably responsible for these effects. Therefore, we undertook a post hoc search to elucidate the link between arecoline, AChRs, and oral cancer.

Previous research has convincingly demonstrated that both arecoline and guvacoline activate muscarinic acetylcholine receptors 1 and 3 (M1 and M3 mAChRs) [85], while only arecoline produces significant activation of the α4 nicotinic receptor and acts as a silent agonist of α7 nAChR [86]. A molecular docking simulation and antagonist co-exposure experiments also showed that arecoline has a strong affinity to muscarinic receptors M1–M4 [87]. Hence, it is likely that arecoline elicits cholinergic signals in the oral mucosa via the M2, M3, and M4 mAChR subtypes that are expressed in oral keratinocytes [88]. Importantly, muscarinic receptors work synergistically with nicotinic receptors to regulate keratinocyte adhesion, most probably by modulating cadherin and catenin levels and activities [89]. Given that alpha 3, alpha 5, alpha 7, and beta 2, as well as the alpha 9 nAChR subunits, are expressed in oral keratinocytes [88], a non-neuronal cholinergic system of the oral mucosa exists that regulates key biological functions such as cell viability, proliferation, migration, adhesion, terminal differentiation, and the secretion of cytokines and growth factors [90].

This keratinocyte cholinergic system has been shown to play a role in oral mucosal diseases [91] and also mediates nicotine toxicity in oral keratinocytes and in epithelial cancers [92]. It is now known that the nAChRs expressed on the cell membrane and mitochondria mediate both growth-promoting and anti-apoptotic effects synergistically. Other mechanisms associated with nicotine toxicity include the genotoxic action of reactive oxygen species [93]. With regard to mAChRs, accumulating evidence suggests that mAChR-dependent signaling pathways can promote cell proliferation and cancer progression [94]. In particular, previous experimental results indicated that M3 receptor activation may promote malignancy in epithelial cancers. In one example, M3-deficient mice displayed reduced epithelial cell proliferation and decreased tumor number and size in models of intestinal neoplasia [95,96]. Similarly, M1 receptor deficiency inhibited mAChR-mediated prostate cancer invasion and metastasis in mouse models of prostate cancer [97].

In summary, there is increasing evidence that the non-neuronal cholinergic system in epithelial tissues is involved in carcinogenesis. Similar to the effects of nicotine, it is reasonable to speculate that AChR ligands, such as arecoline and other areca alkaloids, induce pro-tumorigenic effects in the oral mucosa via receptor-mediated signaling.

## 3. Discussion

In the present study, we employed a theory-driven interpretive review methodology to inform, extend, and supplement the conventional systematic review workflow. This approach is particularly useful when attempting to make sense of heterogeneous evidence in diverse contexts, in a similar manner to that employed in realistic reviews [98]. The results identified cell proliferation, invasion, adhesion, and migration, increased collagen deposition and fibrosis, and altered the immune, inflammatory and epigenetic mechanisms as important events in the pathogenesis of arecoline-induced OSF and OSCC. We generated a framework whereby AChRs were putatively identified as important mediators of these pathophysiological processes.

The initial scoping review identified 90 direct evidence-based primary studies that addressed arecoline-induced carcinogenesis in oral tissues and the potential diagnostic markers associated with the process, all published between 1994 and 2023. All the primary in vivo studies involved the use of murine models challenged with arecoline, while the in vitro studies used a range of human oral cell cultures, such as HOKs or OSCC cell lines that were challenged with arecoline. The subsequent iteration of the scoping review, with a refined search string focused on AChRs, only retrieved one study. Despite this limited result, we collected substantial evidence to show that arecoline activates the local cholinergic axis via AChRs. These receptors are expressed in the oral mucosa and are known to mediate the pro-carcinogenic effects of nicotine [93]. Given that arecoline binds to the same receptor family as nicotine, albeit with a preferential affinity to mAChRs, it is not unreasonable that this areca alkaloid promotes oral carcinogenesis in a similar fashion to nicotine. Hence, we propose that future research should focus on this conceptual framework.

Most of the in vivo studies examined in this review directly challenged mice with arecoline and/or its metabolites, whereas 3 studies injected arecoline-induced OSCC cell lines into their BALB/c nude mice.

These three studies demonstrated the pro-fibrotic property of arecoline in stimulating collagen deposition in the oral mucosa of mice [8,19,49]. TGF-β, the key player in fibrosis, was shown to increase in response to arecoline exposure [12,21,48]. In one particular study, the injection of arecoline into the buccal mucosa of mice directly induced the OSF associated with the DEC1/FAK/Akt signaling pathway [49]. As AChRs are expressed in oral fibroblasts [99], it is plausible that the effect of arecoline on this cell type may involve receptor-mediated mechanisms that may be involved in the pathogenesis of OSF.

Arecoline, despite having cytotoxic effects at higher concentrations, was observed to induce squamous cell hyperplasia when injected into mouse models [8,18,19,20], suggesting that arecoline possesses cellular proliferative properties. Furthermore, studies into gene expression revealed that arecoline upregulates NOTCH1, PTK6, and DDR1 [8,10,12,22], which promotes sustained growth signals while suppressing the tumor suppressor signal RARB [23], allowing cells to evade the growth suppressors.

One study observed that arecoline exposure increased HSP27, an antioxidant protein, suggesting that arecoline could play a role in the cellular adaptation and survival of tumor cells in the presence of oxidative stress [50].

Arecoline was also found to promote invasion. In one study, injecting mice with arecoline-stimulated OSCC resulted in a 50% increase in mice with cervical LN metastasis [48]. Activation of the invasion property in epithelial cells is associated with EMT. Several factors and signaling pathways have been implicated in promoting EMT in mouse models, including TGF-β signaling, DEC1 upregulation leading to FAK/Akt activation [49], PTk6 expression with E-cad suppression [10,27,34], and Krt17 upregulation [52]. Pertinently, the role of AChRs in invasion, migration, and metastasis is well established [100,101].

Analysis of the in vitro research yielded conflicting results. Studies showed that arecoline exposure led to increased or uncontrolled cellular proliferation and tumor cell growth, via the upregulation of oncogenes such as PCNA, Ki67, MEK1, ERK, B-Raf proto-oncogene, serine/threonine kinase (*BRAF*), ZEB1, FAT atypical cadherin 1 (FAT1), NOTCH1 via increased IL-1β, CYR61, and lysine-specific demethylase 1 (LSD1), while simultaneously downregulating the expression of tumor suppressor genes such as tumor proteins 53, 21, and 27 (p53, p21, p27), DUSP4, and the more upstream activators of p53 such as the maternally expressed 3 gene (*MEG3*) [8,24,31,32,33,34,36,43,53,55,56,70]. Generally, arecoline increases the proportion of mitotic cells and could arrest cells at the prometaphase, resulting in misaligned chromosomes such as cyclin B1 [37]. However, conflicting results were found in relation to arecoline’s effects on cell cycle arrest and apoptosis. Some studies reported increased cellular proliferation and the inhibition of apoptosis in oral keratinocytes, while others found that arecoline promoted apoptosis and cell-cycle arrest in the G1/S phase and reduced cellular proliferation in SAS cancer cells and epithelial cells, as well as increasing the expression of *ADHFE1* and *ALDH1A2,* and increased the phosphorylation of Chk1 and Chk2 [26,32,39,40,41,42]. These contrasting results are probably due to the different arecoline concentrations and cell lines used. Increased DNA damage was commonly observed in multiple studies through increased γH2AX and increased 53BP1, which are biomarkers for double-stranded DNA breaks [19,24,45,79,80,102]. Interestingly, DNA repair was often increased initially in the cells and then subsequently decreased as the exposure time and dose of arecoline increased [24,69], which was observed through the reduced phosphorylated ataxia telangiectasia–mutated (p-ATM) gene level [24,46] and increased miR-23a expression [82].

EMT has been shown to play a major role in carcinogenesis; our study highlighted the finding that arecoline directly promotes EMT in a dose-dependent manner in oral epithelial cells via specific factors and multiple signaling pathways, including TWIST overexpression leading to the loss of E-cad [27,62,79], DEC1/FAK/AKT pathway upregulation [49], PA28γ leading to the BRAF/MEK1/ERK pathway [53], and snail family *SNAI1* expression [54], which downstream leads to enhanced EMT. The biomarkers for EMT, such as the increased expression of N-cadherin and vimentin, were found to be elevated as a result of arecoline exposure in multiple studies [13,27,34]. Arecoline was also found to alter cellular adhesion, which is paramount in carcinogenesis, via the upregulation of αvβ6 integrin expression in HOK and the increased attachment of U397 mononuclear cells to EAhy926 cells [57,58]. Arecoline was found to promote cellular migration by upregulating S100A4, Lin28B, and COX-2 expression in OSFs and OE cells [25,59,60,61]. EMT is associated with the acquisition of invasion and metastasis. Clinical markers for predicting lymph node spread and metastasis, such as Lin28B and HSP47, were increased in OSCC compared to normal epithelium in a dose- and time-dependent manner related to arecoline exposure [25,63]. It is possible that these effects are mediated by the local cholinergic system as there is abundant evidence in the literature to show that AChRs control cell–cell cohesion and EMT in the keratinocytes [103,104,105]. These effects are probably pleiotropic in epithelial cells as nicotine has been shown to induce proliferation, invasion, and EMT in a variety of human cancer cell lines, including breast, lung, and pancreatic cells [106].

The link between arecoline, fibrosis, and OSF was also explored. Arecoline is linked to fibrosis, a condition that causes the lining of the mouth to become thick and fibrous. Multiple in vitro studies have explored the potential underlying mechanisms and signaling pathways, including TGF-β signaling, the downregulation of the c terminus of Hsp70 interacting protein (CHIP), leading to increased myofibroblast activities [66], α-SMA and MAD proteins (SMAD) [55,57,66], miR-10b upregulation [79], TWIST expression [62,79], early growth response-1 (EGR-1) expression, leading to Wnt5a activation [41], TIMP-1 and TIMP-2 upregulation leading to inhibition of matrix metalloproteinases [42,46,47], and altered MMPs expression [42,45,47,64]. These findings were suggested as showing the upregulation of transdifferentiation and the activity of myofibroblasts, contributing to increased collagen deposition and fibrosis. This possibly accounts for the onset of OSF, which is associated with a high risk of progression to cancer. Arecoline-treated BMFs resulted in the increased expression of various fibrosis markers, such as PAI-1, and elevated ECM synthesis and secretion through the upregulation of S100A4 [47,60,61,63,64,65]. Arecoline-induced fibrosis is often associated with micro-hypoxia, which was observed through an increased CAIX level [44].

The production of ROS and inflammatory cytokines are major contributors to carcinogenesis. Arecoline has been shown to induce PGE2, IL6, IFN-y, and TNF-a production by oral keratinocytes, inducing inflammation [8,77,78]. In another study, it was implicated in the adaptive immune response by increasing Th17 and decreasing T-reg T-lymphocyte pathways, linking to a potential dysregulated immune response [107]. Arecoline also induced immune evasion in tumor cells in one study, where PD-L1 was upregulated in cancer cells [28]. nROS production was increased in multiple studies when cells were challenged with arecoline or its metabolites [12,26,54,68,69]. In another study, arecoline upregulates CYP26B1, which is thought to play a role in arecoline catalysis, potentially being associated with ROS production [41]. In relation to these findings, some studies showed a concordant decrease in the antioxidant activities of cells challenged by arecoline, such as a significant reduction in catalase [12,69]. Meanwhile, others found an increase in activity [64,70,72,73,74], for instance, in increased heme oxygenase-1 enzyme (HO1); however, this could play a role in enhancing the cell survival of tumor cells against oxidative injury. Most of our studies found the elevated expression of several inflammatory cytokines such as TGF-β, but one study observed a reduction in IL-6 [8,12,19,45,48,57,75,76,77,78]. In this regard, the relationship between oxidative stress and AChRs is well-established, although the prevailing literature suggests that acetylcholine suppresses oxidative stress-mediated pathways [108,109,110].

Finally, various miRNAs in cells have been shown to either reduce or increase in response to arecoline exposure. These miRNA changes have been shown to lead to increased DNA damage, increased cellular proliferation, increased cellular invasion, and cellular migration [31,42,76,82]. A recent study showed that 10 long noncoding RNAs (lncRNAs) that were dysregulated by areca nut were also altered in HNC patients [111]. Of these, five oncogenic (lung cancer associate transcript 1 (*LUCAT1*), MIR31 host gene (*MIR31HG*), urothelial cancer-associated 1 *(UCA1*), hypoxia-inducible factor 1A antisense RNA 2 (HIF1A-AS2), and SUMO1 pseudogene 3 (*SUMO1P3*)) and tumor-suppressive long intergenic non-protein coding RNA 312 (*LINC00312*) were independently validated, thereby identifying solid lncRNA signatures that play a role in areca nut-induced HNC [111]. In multiple studies, arecoline was shown to downregulate tumor suppressor activities, such as reduced miR-22, miR211-TCF12, miR329, miR410, miR-379, miR-30a, miR483-5p, and miR-886-3p and to upregulate the proto-oncogenes ADHFE1, ALDH1A2, and DDR-1, which are associated with increased cell proliferation and anti-apoptosis [22,31,32,51,76,81]. In many studies, various miRNAs are upstream upregulators of proto-oncogenes. For instance, in OSCC tissues, miR483-3p expression was down-regulated and DDR1 was upregulated, which was associated with lymph node metastasis [22]. The downregulation of *miR329* and *miR410* will result in WNT-7b over-expression, which activates the WNTb catenin pathway, thereby promoting proliferation and invasion [31]. Reduced miR200c levels are associated with the increased transdifferentiation of myofibroblasts, leading to fibrosis [83].

While our iterative review process was aimed at a scoping review of original studies addressing the molecular mechanisms of arecoline-induced carcinogenesis, we note that several systematic reviews on this topic have been published. Ko et al. linked arecoline and ANO to an increase in the expression of EMT inducers, such as reactive oxygen species, TGF-β1, NOTCH1, and inflammatory cytokines, and to the activation of EMT-related proteins [112]. Our mixed-methods approach is original in that it used the current knowledge base to identify gaps in the literature and inform further theory-driven searches that allowed us to identify potentially crucial mechanisms in OSF pathophysiology.

As with all study designs, our interpretive review does not come without its limitations. Our data synthesis was derived from only two relevant databases, which means that we could potentially have missed relevant studies in other databases. No clinical studies could be included, as there were no studies that tested the oral carcinogenic effect of arecoline in isolation (i.e., not in the context of areca nut or betel quid) on live humans. It should be noted that the vast majority of the included studies were from either Taiwan or India, so the sample and researcher variations are low and some systematic biases might have been introduced. Although in vivo animal models are invaluable in studying oral carcinogenesis, the results may be neither reliable nor applicable to human tissues, and most studies had a relatively small sample size. Finally, there was heterogeneity of data, with a wide breadth of signatures being implicated in carcinogenesis and conflicting results regarding their respective mechanisms.

## 4. Materials and Methods

### 4.1. Study Design

The iterative review process included four phases: (1) a scoping review, including qualitative synthesis of the results; (2) refinement of the search strategy, based on selected themes and/or relevant adjacent literature; (3) a second round of scoping review complemented by (4) a post hoc manual search, leading to the generation of key concepts.

### 4.2. Scoping Review Methodology

The scoping reviews were conducted in accordance with PRISMA-ScR guidelines [17]. The following databases were searched: PubMed and the Web of Science.

The research questions being addressed in the initial scoping were: What are the molecular mechanisms underlying arecoline-induced oral carcinogenesis? Which specific signatures are associated with arecoline-induced oral carcinogenesis and how do they contribute to disease pathogenesis?

To comprehensively screen a wide range of studies, the search strategy incorporated terms such as “cancer”, “carcinoma”, and “carcinogenesis”. Given the interest in oral cancer, the search was confined to terms related to the “mouth”, “oral”, or “oral cavity” search terms. The search strategy encompassed the “diagnostic markers” associated with oral carcinogenesis and terms relevant to “pathogenesis”.

There were three main concepts in our initial search strategy:

1. The condition: cancer;
2. Etiology of the condition: arecoline;
3. Location of interest: oral.

The following search string and related terms were used to find the relevant publications: (cancer OR carcinogenesis OR carcinoma) AND (mouth OR oral OR oral cavity) AND (arecoline) AND (pathogenesis OR diagnostic markers).

The second round of searches included the following string and related terms: (cancer OR carcinogenesis OR carcinoma) AND (mouth OR oral OR oral cavity) AND (arecoline) AND (acetylcholine OR AChR*).

There were no restrictions on the publication dates within the searched databases. Publications released up until April 2023 were included in this review.

Although additional keywords such as “OSCC” and “biomarkers” were included in the pilot calibration, they did not significantly alter the search results. The inclusion of the terms “areca nut” and “betel nut” did yield a notable increase in the number of articles retrieved. However, for the purpose of this study, the focus was specifically on the role of arecoline (and its metabolites) in the mechanism of carcinogenesis, excluding the broader context of betel nut. Therefore, the search string was considered comprehensive enough to encompass the various terminologies related to oral carcinogenesis and to specifically target the involvement of arecoline as the etiological factor.

During the review process, careful consideration was given to the inclusion and exclusion criteria. Despite our focus on cancer, studies reporting on OSF were included in this review, as we believe that these could be relevant to understanding the mechanisms of oral carcinogenesis. Studies that were not published in the English language, as well as reviews (systematic and meta-analyses), book chapters, and non-peer-reviewed literature, were manually excluded. We excluded those studies that did not test arecoline in isolation, those that conducted an investigation on cells derived from outside of the oral cavity, and studies with indirect evidence drawn from different treatment modalities. Duplicate articles were manually excluded by comparing the search results from PubMed and the Web of Science.

### 4.3. Data Screening

All reviewers evaluated the same set of 196 publications that were obtained from PubMed and the Web of Science. Through discussions, the screening process was refined for this review. The data screening process was rigorous, involving five independent reviewers who meticulously assessed the titles and abstracts of the articles while following predetermined exclusion criteria. For articles that were initially unclear in terms of eligibility, a thorough examination of the full text was conducted to determine their inclusion status. Disagreements regarding study selection and data extraction were resolved by means of consensus among the reviewers. Inter-rater agreement was measured using the kappa score, and any disagreements were resolved by an impartial third-party adjudicator. The inter-rater agreement was 89.9%.

### 4.4. Data Extraction and Synthesis

To ensure consistency and standardization, a pre-established data extraction sheet was utilized to extract the relevant information from the full texts of the eligible studies.

The evidence obtained from the selected studies was presented in two ways. Firstly, the data extracted from the final search were organized and presented in the form of extraction tables. These tables included details such as the study type, population/sample size, intervention and control groups, outcomes, cell culture/chemical investigations, the compounds used, and diagnostic markers, as well as the key findings and conclusions. The tables presented in this paper followed a PICO format for brevity and visual clarity.

Secondly, a narrative component was integrated into the results section, providing a comprehensive overview of the investigations and consistently reporting the outcomes across multiple studies.

## 5. Conclusions

This critical interpretive synthesis enabled the identification of prevailing trends and the consolidation of data pertaining to the molecular mechanisms involved in carcinogenesis, while also highlighting crucial gaps and inconsistencies in the literature. The iterative review process and the final synthesis allowed for possible explanations to be found for arecoline-induced carcinogenesis.

In our scoping reviews, we discussed the molecular mechanisms by which arecoline promotes OSF and OSCC and recognized a wide range of specific diagnostic markers implicated in arecoline-induced carcinogenesis. We were able to highlight common mechanisms in the pathogenesis of disease, including pro-fibrotic effects, increases in DNA damage and apoptosis, increased cellular proliferation, and EMT promotion, from both in vivo and in vitro studies. This comprehensive overview of the diagnostic and prognostic biomarkers implicated in OSF carcinogenesis can guide future research directions toward evaluating diagnostic potential and accuracy and, thus, aid earlier detection and intervention.

Nonetheless, the data extracted was heterogeneous and inconsistencies were observed, necessitating a further post hoc search. The results show that most of the mechanisms that are potentially responsible for the detrimental effects of arecoline in the oral cavity may be explained by the activation of the local cholinergic system via arecoline–AChR binding.

With the results of our review, we can present insight into the most promising molecular targets in arecoline-induced OSF and OSCC, which can inform future research into topics such as the downstream effects of AChR-mediated pathways and the development of potential preventative and therapeutic options.

## Supplementary Materials

The following supporting information can be downloaded at: https://www.mdpi.com/article/10.3390/ph16121684/s1, Table S1: Summary of the in vitro studies included in the initial scoping review [113,114,115,116,117,118,119,120,121].

## Abbreviations

Abbreviation	Definition
AChR	acetylcholine receptors (m: muscarinic)
*ADHFE1*	alcohol dehydrogenase iron containing 1
*ALDH1A2*	aldehyde dehydrogenase 1 family member A2
ANO	arecoline N-oxide
α7-nAChR	alpha-7-nicotinic acetylcholine receptor
a-SMA	alpha smooth muscle actin
BALB/c mice	albino laboratory-bred mice
BMF	buccal mucosa fibroblasts
BQ	betel quid
*BRAF*	B-Raf proto-oncogene, serine/threonine kinase
CAIX	carbonic anhydrase IX
CAL27	epithelial squamous cell carcinoma cell line
CAL-33	cellosaurus squamous cell carcinoma cell line
*CASP8*	caspase 8
CHIP	c terminus of the Hsp70 interacting protein
Chk	checkpoint kinase
*CYR61*	cysteine-rich angiogenic inducer 61
C57BL/6	a laboratory-bred strain of mice
*DDR1*	discoidin domain receptor 1
1-Dec	deleted in esophageal cancer RNA gene
*DUSP4*	dual specificity phosphatase 4
E-cad	E-cadherin
ECM	extracellular matrix
*EGFR/Pl3k*	epidermal growth factor receptor/phosphoinositide 3-kinases
EMT	epithelial–mesenchymal transition
FAK/AKT	focal adhesion kinase/serine/threonine kinase
FAT1	FAT atypical cadherin 1
FAM213A	Peroxiredoxin-like 2A
FOXD3	forkhead box D3
FTO	fat mass and obesity-associated gene
HNSCC	head and neck squamous cell carcinoma
HO1	heme oxygenase-1 enzyme
HOK	human oral keratinocyte
HSP	heat shock protein
HES1	hairy enhancer of split 1
HIF1A-AS2	hypoxia-inducible factor 1A antisense RNA 2
IGF-R1	insulin-like growth factor receptor 1
IL	interleukins
IFN-γ	interferon gamma
Ki67	Kiel 67
Krt17	keratin 17
Lin28B	Lin-28 homolog B
*LINC00312*	tumor-suppressive long intergenic non-protein coding RNA 312
LN	lymph node
LSD1	lysine-specific demethylase 1
*LUCAT1*	lung cancer associate transcript 1
*MEG3*	maternally expressed 3 gene
MEK1/ERK pathway	mitogen-activated protein kinase 1/extracellular signal-regulated kinase pathway
MGMT	O6-methyl-guanine-DNA methyltransferase
miRNA	microRNA
*MIR31HG*	MIR31 host gene
MMP	matrix metalloproteinase
*MYC*	MYC proto-oncogene
NFκB	nuclear factor kappa B
NNK	nicotine-derived nitrosamine ketone
NOD/SCID	non-obese diabetic homozygous for the SCID mutation with immune impairment
NOM	N-oxide mercapturic acid
*NOTCH1*	notch receptor 1
OE	oral epithelial cell
OECM-1	human oral squamous carcinoma cell line
OK	oral keratinocyte
OSCC	oral squamous cell carcinoma
OSF	oral submucous fibrosis
OSM	oncostatin M protein
PAI-1	plasminogen activator inhibitor-1
pAKT	phosphorylated AKT serine/threonine kinase 1
p-ATM	phosphorylated ataxia telangiectasia–mutated
PA28γ	proteasome activator complex subunit 3
PCNA	proliferating cell nuclear antigen
PGE2	prostaglandin E2
PDL1	programmed death-ligand 1
PLC/IP3/Ca^2+^/calmodulin	phospholipase C/inositol triphosphate 3/calcium/calmodulin
*PRDX2*	peroxiredoxin 2
*PTK6*	protein tyrosine kinase 6
*PTPRM*	protein tyrosine phosphatase receptor type M
p21	cyclin-dependent kinase inhibitor 1
p27	cyclin-dependent kinase inhibitor 1B
*RARB*	retinoic acid receptor beta
ROS	reactive oxygen species
Rho	ras homologous protein family
*SAA1*	serum amyloid A1
SIRT1	sirtuin 1
SMAD	α-SMA and MAD proteins
*SNAI1*	zinc finger protein 1
*SUMO1P3*	SUMO1 pseudogene 3
*S100A4*	S100 calcium binding protein A4
*TCF12*	transcription factor 12
Tg	transgenic mouse line
TGF-β	transforming growth factor-β
TIMP	tissue inhibitor of metalloproteinase
TNF-α	tumor necrosis factor alpha
*TP53*	tumor protein p53
*WNT*	Wnt pathway
*UCA1*	urothelial cancer associated 1
*ZEB1*	zinc-finger E box-binding homeobox 1
4-NQO	4-nitroquinoline 1-oxide
53BP1	p53-binding protein 1
γH2AX	phosphorylation of the H2AX variant histone
