# Supplementary material for: A Critical Interpretive Synthesis of the Role of Arecoline in Oral Carcinogenesis: Is the Local Cholinergic Axis a Missing Link in Disease Pathophysiology?
Source: Pharmaceuticals (Basel). 2023 Dec 4;16(12):1684. doi: 10.3390/ph16121684 (PMC10748297; doi:10.3390/ph16121684)
Supplement: Supplementary file 1 [file pharmaceuticals-16-01684-s001.zip › pharmaceuticals-2682569-supplementary.pdf]

Supplementary Table S1. Summary of *in vitro* studies included in the initial scoping review.

**Author, Year**	**Population/Model/ Intervention**	**Outcomes/Diagnostic Markers Measured**	**Results Observed**
Ren et al., 2021 [48]	OSCC cell lines (CAL33, UM2) were challenged with arecoline	Histopathological changes; pro-inflammatory cytokines (SAA1, IL, CCL, TNF)	Arecoline induced EMT, cell migration and invasion<br><br>Arecoline induced elevation of SAA1, IL1A, IL36G, CCL2, CCL20, and TNF levels
Nithiyanantham et al., 2021 [12]	OSCC cell lines (SAS, HSC-3, OC-3) were challenged with arecoline and ANO	Antioxidants (glutathione, catalase, NAC), ROS, NOTCH1, cytokines (IL1B, IL17a), DNA damage marker	Arecoline and ANO significantly reduced antioxidants, induce d ROS, NOTCH1, IL1B, IL17a levels, and elevated DNA damage
Li et al., 2022 [21]	OSCC cell lines (CAL27 and SCC25) treated with low-dose arecoline.	Fat mass and obesity-associated protein (FTO), MYC, programmed cell death-ligand (PD-L1)	Arecoline induced upregulation of FTO, which then increases expression of MYC and PDL1.
Kuo et al., 2019 [8]	OSCC cell lines (HSC-3, SCC9), normal human gingival fibroblast (HGF1) were challenged with ANO	NOTCH1, FAT1, cytokines (IL-1B, TNF-a), proliferating cell markers (PCNA, Ki67), DNA damage marker (Gamma H2AX)	ANO induced elevation of NOTCH1, FAT1, IL-1B, TNF-a, cell proliferation, and DNA damage
Li et al., 2022 [28]	Human keratinocyte (HaCat) were challenged with arecoline	TGF-B, Mettl3, Mettl4	Arecoline induced elevation of TGF-B, Mettl3, and Mettl4 levels
Wang et al., 2022 [113]	OSCC cell lines (SCC25, CAL27) were challenged with arecoline	Mettl3, HIF-1a	Arecoline induced elevation of Mettl3 and HIF-1a levels
Li et al., 2021 [21]	OSCC cell lines (SCC25, SCC9, HSC-2, CAL27) were	FTO, Mettl3, Mettl4, FOXA2	Arecoline induced elevation of FTO and Mettl3 levels but

	challenged with arecoline		reduced FOX2A expression
Tu et al., 2019 [24]	OSCC cell lines (OEC-M1, SAS) and gingival epithelial SG cells were challenged with arecoline	Proliferating cell marker (PCNA), DNA damage marker (gamma H2AX), DNA repair enzymes (p-ATM)	Arecoline induced elevation of cell proliferation, DNA damage, DNA repair (however DNA repair is reduced with longer duration of arecoline)
Hsieh et al., 2022 [9]	OSCC cell lines (OCMC, OECM1, OML3, OCSL) were challenged with different conditions: PTK6 overexpression (induced by arecoline) and knockdown	Proliferating rate, migration and invasion abilities	Arecoline-induced PTK6 overexpression increased proliferation rate, migration, and invasion
Yang et al., 2021 [114]	Normal buccal mucosal fibroblasts (BMFs) and OSF tissues were challenged with arecoline	MiR21	Arecoline induced elevation of miR21 in a dose-dependent manner
Shih et al., 2021 [69]	OSCC cell line (OEC-M1) were challenged with arecoline	ROS, antioxidant (catalase), DNA damage, DNA repair (p-ATM), TGF-B	Arecoline induced cytotoxicity, increased ROS production, and DNA damage<br><br>Arecoline reduced DNA repair and antioxidant
Kuo et al., 2015 [19]	Normal human gingival fibroblast cells (HGF1, CRL-2014) and normal human oral keratinocyte (HOK) were challenged with arecoline or ANO	Cytotoxicity (via lactate dehydrogenase release), collagen 1, TGF-B, DNA damage (gamma H2AX)	Arecoline and ANO Induced cytotoxicity and elevated collagen 1, TGF-B, and DNA damage
Wang et al., 2016 [13]	Human oral epithelial cell lines (SG, FaDu)	EMT property, miR-145	Arecoline induced EMT but reduced miR-145 level

	were challenged with arecoline		
Hu et al., 2022 [49]	Human oral keratinocytes (HOK) were challenged with arecoline	DEC1/FAK/Akt, EMT (E-cadherin)	Arecoline induced elevation of DEC1, FAK, Akt in a dose-dependent manner and induced EMT
Chuerduangphui et al., 2020 [29]	OSCC cell lines (ORL-48T, ORL-136T) and human tongue keratinocytes (HTK-K4DT) were challenged with arecoline	PRDX2	Arecoline induced elevation of PRDX2
Shiah et al., 2020 [32]	OSCC cell lines (DOK) were challenged with arecoline	Retinoid signalling related genes (ADHFE1, ALDH1A2), DNMT3B, miR30a, miR379	Arecoline reduced expression of retinoid signalling related genes, miR30a, and miR379.<br><br>Arecoline induced elevation of DNMT3B
Lee et al., 2016 [62]	Human primary buccal mucosal fibroblasts (BMFs) were challenged with arecoline	Twist	Arecoline induced elevation of Twist expression in a dose-dependent manner
Chuerduangphuet al., 2018 [76]	Human OSCC cell lines ORL-48(T) and ORL-136(T) were challenged with arecoline	c-Myc, IL-6, STAT3, miR-22, OSM	Arecoline induced elevation of IL-6 and STAT3 proteins<br><br>Arecoline induced c-Myc and OSM expression at both the mRNA and protein levels<br><br>Arecoline reduced miR-22 expression
Chang et al., 2017 [20]	Human dysplastic oral keratinocyte cell (DOK) and CAL27	CASP8	Arecoline induced CASP8 mRNA expression and

	(ATCC CRL2095) cell lines were challenged with ANO		proteins in both DOK and CAL27 cell lines
Yang et al., 2014 [44]	Human buccal mucosal fibroblasts were challenged with arecoline	CAIX	Arecoline induced elevation of CAIX expression in a dose-dependent manner
Xie et al., 2022 [53]	Two epithelial cell lines, HOK16E6E7 (HOK) and HaCat, were challenged with arecoline	PA28γ, MEK1, p-MEK1, p-ERK, BRAF	Arecoline induced elevation of PA28γ, p-MEK1, p-ERK, and BRAF levels
Fang et al., 2019 [11]	Normal human mucosa were challenged with arecoline	Slug	Arecoline increased Slug expression in normal BMFs.
Lu et al., 2018 [83]	Normal buccal mucosal fibroblasts were challenged with arecoline	miR-200c	Arecoline reduced the relative expression of miR-200c in a dose-dependent manner in normal BMFs
Chen et al., 2016 [51]	OSCC cell lines SAS, OECM1, HSC3, FaDu, OC4, and SCC25; 293T cells and phoenix package cells; and six primary OSCC cells isolated from different tumours were challenged with 4NQO and arecoline	miR-211	4NQO and arecoline upregulated miR-211 expression in OSCC cells
Li et al., 2023 [2]	Human tongue squamous cancer cells (CAL27) and human primary buccal mucosal fibroblasts (NFs) were challenged with arecoline.	Cell proliferation, DNA (genes)	Arecoline promoted cell proliferation in a dose dependent manner in both NFs and OSCC cells.<br><br>Arecoline induced differentially expressed genes (DEG).

			Common DEGs in both NFs and CAL27 cells after being treated with arecoline were identified, including cyclin-dependent kinase-like 1 (CDKL1), mitochondrial ribosomal protein L12 (MRPL12), and ribosomal RNAs (RNA5S9, RN7SL4P).<br><br>Arecoline induced genome-wide methylation levels in both CAL27 cells and NFs.<br><br>Key methylated genes induced by arecoline. were identified, including PTPRM and FOXD3 in NFs, SALL3 and IRF8 in OSCC cells.
Deng et al., 2011 [70]	Human normal gingival epithelial Smulow–Glickman cells were challenged with arecoline	Cyr61	Arecoline induced elevation of Cyr61 expression in a dose-dependent manner
Chang et al., 2014 [55]	Normal human buccal mucosal fibroblasts were challenged with arecoline	ZEB1, a-SMA	Arecoline induced a-SMA, and ZEB1 expression in BMFs.
Chen et al., 2020 [30]	Human oral fibroblasts exposed to arecoline	Egr1 and Wnt5a	Arecoline induced Egr1 and Wnt5a expression. This is associated with promotion of fibroblast proliferation.

Chen et al., 2014 [41]	Human gingival fibroblasts (HGF) and oral epidermal gingival squamous carcinoma (Ca9-22 cell line) were challenged with arecoline	MAO-A	Arecoline induced elevation of MAO-A expression in HGF cells and downregulation of MAO-A expression in Ca9-22 cancer cell line
Hu et al., 2015 [60]	Oral epithelial cells (SG and OECM1) were challenged with arecoline	S100A4	Arecoline induced elevation of S100A4 expression in a dose-dependent manner in oral epithelial cells
Zheng et al., 2018 [34]	Human oral submucosal fibrosis tissues and normal oral mucous - primary oral mucosal fibroblasts were treated with arecoline alone or co-treated with arecoline and TSN.	LSD1, p53, p21, puma, E-cadherin, N-cadherin, and vimentin	Arecoline treatment decreases p53 and its downstream molecules in oral mucosal fibroblasts, but TSN reverses this effect dose-dependently by inhibiting LSD1 and reversing TP53 promoter hypermethylation. TSN also counteracts the effects of arecoline on LSD1, p53, p21, puma, E-cadherin, N-cadherin, and vimentin expression.
Chou et al., 2019 [22]	OKF4/hTERT, OEC-M1, and TW2.6 OSCC cell lines were challenged with arecoline	DDR1, miR-486-3p	Arecoline increased DNMT3B, but not DNMT3A.<br><br>Arecoline attenuated the expression of miR-486-3p in OSCC.<br><br>Arecoline treatment decreased the expression level of ANK1 mRNA and miR-486-3p and increased the DDR1

			mRNA and protein level in OKF4/ hTERT (an immortalized normal oral keratinocytes cells). Arecoline treatment increased the cellular proliferation in OKF4/hTERT cells.
Shiah et al., 2014 [31]	OSCC cells, including DOK, FaDu, OC-3, OEC-M1, SCC-4, SCC-9, SCC-15, SCC-25, Tw2.6, and YD-15 were challenged with arecoline	miR329, miR410, Meg3, Wnt-7b	Arecoline reduced miR329, miR410 and Meg3 expression. Silencing of miR329 and miR410 activate the Wnt–b-catenin signalling pathway and promote proliferation and invasion in OSCC.
Fang et al., 2020 [79]	Normal buccal mucosal fibroblasts (BMFs) were challenged with arecoline	miR-10b	Expression of miR-10b was induced in arecoline-treated normal BMFs in a dose-dependent manner
Islam et al., 2020 [115]	Human gingival epithelial progenitors (HGEPs) were challenged with arecoline.	SIRT1	The methylation level of SIRT1 in cells challenged with arecoline was significantly increased.<br><br>The expression levels of SIRT1 mRNA in the cells challenged with arecoline were significantly decreased
Jeng et al., 2003 [77]	Oral KB carcinoma cells and primary gingival keratinocytes (GK) were challenged with arecoline	PGE2, IL-6, TNF-a	Arecoline stimulated PGE2 in a dose-dependent manner; arecoline markedly decreased IL-6 production; arecolin

			e showed little effect on TNF-a production
Wang et al., 2020 [107]	Normal human buccal fibroblasts were challenged with arecoline	Th17, Treg	Arecoline led to the upregulation of Th17 and downregulation of Treg
Lee et al., 2011 [64]	OSCC cell line OC2 cells were challenged with arecoline	HSP47	Arecoline was found to elevate HSP47 expression in a dose- and time-dependent manner
Ji et al., 2012 [43]	Two OSCC cell lines, OCSL and OC2 were challenged with arecoline	p21, p27	Arecoline downregulated p21 and p27 in OSCC cells with high confluence
Adhikari et al., 2021 [33]	Human gingival epithelial progenitors with challenged with arecoline	DUSP4	DUSP4 was hypermethylated and downregulated following arecoline treatment
Shieh et al., 2003 [46]	OSF buccal mucosa fibroblasts were challenged with arecoline	TIMP-1	Arecoline significantly elevated TIMP-1 protein and mRNA expression
Chang et al., 1998 [80]	Healthy human buccal fibroblasts were challenged with arecoline	Double-stranded polynucleic acid	Arecoline is a cytotoxic agent and no genotoxicity was found in human buccal fibroblasts
Chang et al., 2001 [116]	Oral mucosal fibroblasts (OMF), oral OSCC KB epithelial cells were challenged with arecoline	Cell viability , Δβm, GSH level, intracellular H2O2 production	Arecoline suppressed the growth of KB cells in a dose-dependent manner<br><br>Arecoline led to a depletion of GSH level in KB cells<br><br>Arecoline suppressed H2O2 production in KB cells

			Arecoline led to hyperpolarization of Δβm in KB cells
Yu et al., 2013 [61]	Normal buccal mucosal fibroblasts (BMFs) were challenged with arecoline	S100A4	Arecoline leads to dose- and time-dependent elevation of S100A4 expression in normal buccal mucosa fibroblasts BMFs
Ho et al., 2000 [9]	Normal oral mucosal fibroblasts were challenged with arecoline	C-fos, c-jun	Arecoline induced a three-fold increase in c-jun mRNA levels but did not induce c-fos mRNA expression
Wang et al., 2010 [37]	HEp-2 and OSCC cell line KB cells were challenged with arecoline	Cell morphology, histone H3 at serine 10, aurora A, cyclin B1, Bub1, BubR1, Mad2, Mps1, spindle structures and organisation	Arecoline stabilises mitotic spindle assembly, which leads to distorted organisation of mitotic spindles, misalignment of chromosomes, and upregulation of spindle assembly checkpoint (SAC) genes
Jeng et al., 1994 [35]	Normal human buccal mucosa were challenged with arecoline	Cell viability and proliferation	Arecoline decreases cell survival and proliferation in a dose-dependent manner
Chang et al., 2004 [36]	Human gingival keratinocyte and KB carcinoma cells were challenged with arecoline	IL-6, PGE2, ERK-1/2, c-fos	Arecoline inhibited IL-6 production but stimulated PGE2 production<br><br>Arecoline induced ERK-1/2 phosphorylation

			Arecoline induced c-fos mRNA expression
Jeng et al., 1999 [117]	Healthy gingival keratinocytes were challenged with arecoline	Growth, total DNA synthesis, unscheduled DNA synthesis	Arecoline inhibited growth, reduced total DNA synthesis and unscheduled DNA synthesis in a dose dependent manner
Chang et al., 2001 [118]	Healthy human buccal mucosal fibroblasts were challenged with arecoline	Glutathione-S-transferase activity (GST), TBARS production levels, metabolic activity (alamar blue absorbance)	Arecoline was cytotoxic in a dose dependent manner.<br><br>Arecoline significantly decreased GST activity in a dose dependent manner, but did not increase lipid peroxidation
Chiang et al., 2007 [102]	Human gingival fibroblasts were challenged with arecoline	LDH release assay, whole genome cDNA microarray assay, qRT-PCR validation.	Arecoline was dose-dependently cytotoxic to HGF-1 cell line. Arecoline significantly induces GDF15, HSPA14, DNAJA1, PTGS2 and DDIT4, whilst repressing CHAF1A, CHAF1B, FANCG, BRCA1, S100A12, CYP26B1 and GSS
Chen et al., 2014 [41]	Ca9-22 cells and HGF-1 were challenged with arecoline	Cell viability, CYP26B1 and its splice variant expression	Arecoline reduced cell viability in a dose dependent manner<br><br>Arecoline increased expression of the full length and splice variant of CYP26B1 in both Ca9-22 cells and HGF-1

Moutasim et al., 2011 [57]	OSCC expresses high levels of avB6. avB6-negative OSCC cell lines. Immortalized oral keratinocytes with low avB6 levels (OKF6/TERT-1) challenged with arecoline	avB6 expression, TGF-1 beta activation, SMA, pSmad2, pSmad4, M4 receptor expression.	Arecoline upregulates avB6 expression in keratinocytes modulated by M4 muscarinic receptor, and this activates the TGF-1 beta pathway which promotes myofibroblast transdifferentiation, keratinocyte migration and invasion of OKF6/TERT-1 cells
Lee et al., 2013 [54]	Primary human oral keratinocytes (HOKs) were challenged with arecoline	MGMT expression O6-methyl-guanine-DNA methyltransferase	Arecoline stimulated increased MGMT expression in HOKs
Zhang et al., 2021 [81]	CAL-27 and SCC-4 cell lines were challenged with arecoline	miR-886-3p	Arecoline increased miR-886-3p expression in a dose- and time-dependent manner in both CAL-27 and SCC-4 cell lines
Tsai et al., 2008 [38]	OSCC cell line KB cells were challenged with arecoline	y-H2AX phosphorylation, activation of ATM-dependent signal pathway and cell cycle biomarkers, DNA repair biomarkers, p53 expression and transdifferentiation	Arecoline induced y-H2AX phosphorylation<br><br>Arecoline triggers ATM-dependent pathway and induces G2/M arrest via increasing ATM kinase in KB cells in a dose-dependent manner. Arecoline inhibits expression and transactivation function of p53.
Ho et al., 2015 [56]	Smulow-Glickman human gingival epithelial cell line culture challenged with arecoline	ZEB1 expression, invasion ability and anchorage-dependent growth of cultured cells.	Arecoline induced elevation of ZEB1 in a dose-dependent manner.

Lee et al., 2010 [71]	Oral epithelial cell line (GNM) is challenged with arecoline, curucumin, PD98059, staurosporin.	Hypoxia inducible factor-1. HIF-1a expression.	Arecoline dose-dependently and time-dependently elevated expression of HIF-1a in GNM cells. N-acetyl-L-cysteine (antioxidant), curucumin, PD98059 and staurosporin under cytotoxic concentrations reduced arecoline induced HIF-1a expression significantly.
Lee et al., 2008 [73]	Oral epithelial cell line (GNM) challenged with arecoline	Heme-oxygenase 1 expression	Arecoline dose-dependently elevated HO-1 mRNA in GNM cell cultures. Addition of NAC markedly inhibited arecoline-induced HO-1 expression.
Lin et al., 2015 [25]	Smulow-Glickman human gingival epithelial cell line challenged with arecoline and Lin28B silencing.	Lin28B expression, cell proliferation MTT assay, apoptotic assay, cell invasion analysis	Arecoline dose-dependently increased Lin28B expression in SG human gingival epithelial cells.
Lee et al., 2013 [54]	Primary Human Oral Keratinocytes (HOKs). Oral epithelial cell line OECM-1 challenged with arecoline, NAC, curucumin, EGCG	Cell viability assay of HOKs and OECM-1. ROS generation. expression .	Arecoline induced intracellular ROS generation in HOKs and OECM-1 in a dose dependent manner. Arecoline increased snail expression in a dose and time dependent manner in HOKs and OECM-1. NAC, curucumin and EGCG repressed Arecoline-induced Snail expression.

Tsai et al., 2011 [82]	HGF, Ca9-22 and 293 cells challenged with arecoline	y-H2AX expression, double-stranded breaks repair via plasmid-based assay.	Arecoline induced miR-23a overexpression was correlated with an increase in y-H2AX. Double-stranded break repair was reduced in arecoline treated/miR-23a over-expressed cells.
Rehman et al., 2016 [45]	Early passage oral fibroblast cultures obtained from two donors challenged with arecoline	Cell yield, cellular senescence markers, 53BP1, TGF-B, MMP-2 expression, SA-BGal, 53BPI staining and CDKN2A/P16^INK4A	Arecoline inhibited cell growth and induced cell cycle exit in human oral fibroblasts. Arecoline induced markers of cellular senescence in human oral fibroblasts. Arecoline induced markers of irreparable DNA double-stranded breaks in human oral fibroblasts. Senescent cells induced by arecoline secrete TGF-B and MMP2.
Chen et al., 2020 [66]	Primary normal buccal mucosal fibroblasts (BMFs) and human fibrotic mucosal fibroblasts (fBMFs) from OSF tissue were established and cultivated challenged with arecoline	E3 ligase carboxyl-terminus of Hsp70-interacting protein (CHIP) expression, expression of a-SMA and TGM2.	Arecoline induced myofibroblast transdifferentiation and downregulation of E3 ligase carboxyl-terminus of Hsp70-interacting protein (CHIP) in a dose-dependent manner, and a-SMA and TGM2 levels decreased concordantly
Lee et al., 2008 [73]	Oral epithelial cell line (GNM) challenged with arecoline	Metallothionein-1 (MT-1) expression	Arecoline dose-dependently increased MT-1 mRNA in GNM cells.

Yang et al., 2003 [65]	BMF cell cultures from healthy biopsy specimens challenged with arecoline	PAI-1 mRNA and protein expression.	Arecoline dose-dependently elevated PAI-1 expression in a dose-dependent manner in BMFs.
Liao et al., 2018 [119]	Cell cultures from healthy and fibrotic human oral mucosal biopsies (BMFs and fBMFs respectively) challenged with arecoline	miR-200b expression	Arecoline dose-dependently reduced gene expression of miR-200b in BMFs and fBMFs. Arecoline-induced myofibroblast activities were abolished by overexpression of miR-200b in BMFs and fBMFs.
Chang et al., 2013 [42]	SAS tongue cancer cell cultures. Primary gingival keratinocytes challenged with arecoline	MTT assay for cell viability, Chk1 and Chk2 phosphorylation, TIMP-1 and TMP-2 levels in SAS cell culture.	Arecoline leads to cell death, apoptosis and cell cycle arrest of SAS cells. Arecoline stimulated Chk1 and Chk2 phosphorylation in SAS cells. Arecoline inhibited TIMP-1 and TIMP-2 production in SAS cells.
Yu et al., 2021 [120]	Primary normal buccal mucosal fibroblasts (BMFs) were challenged with arecoline	H19 expression, TGF-B1 levels	Arecoline upregulates H19 expression in a dose-dependent manner in BMFs
Lee et al., 2012 [72]	Normal human oral keratinocytes were challenged with arecoline	HSP27	Arecoline induced elevation of HSP27 expression in a dose- and time-dependent manner
Lee et al., 2008 [64]	OSCC cell lines were challenged with arecoline	MMP-1	Arecoline induced elevation of MMP-1 expression in both mRNA and protein levels

Hsu et al., 2001 [78]	Cultured peripheral blood mononuclear cells from patients with long term betel quid use w/o lesions (N-B) vs normal healthy cells (N) vs cells from OSF patients vs BQ induced OSCC patients (SCC-B) vs non-BQ induced OSCC (SCC-N)patients all challenged with arecoline	IL-2, TNF-, TGF-ß, IFN- cytokine secretion	IL-2: N-B> SCC-B> OSF<br>TNF-: SCC-N> OSF> N-B> SCC-B<br>TGF-: N-B> SCC-B> OSF<br>IFN-: N-B> SCC-B>OSF<br>BQ influences cytokine production by mononuclear cells. Arecoline stimulation in N resulted in overall reduction of IL-2, TNF-, and TGF-ß. IFN- did not change.
Chang et al., 2013 [68]	Human OMF and gingival keratinocytes were challenged with ANE (arecoline and arecaidine)	ANE-induced OMF contraction, ROS production, CES and IP3 receptor expression, exogenous calcium addition, Ca2+-CaM and Rho signaling and MLC and actin filament activation	Arecoline and ANE induce OMF contraction and PLC/ IP3/ Ca2+/ calmodulin, Rho signaling pathway and actin filament polymerisation contribute to ANE-induced OMF contraction
Yu et al., 2016 [121]	Primary human normal buccal mucosal fibroblasts (BMFs) were challenged with arecoline	SSEA-4	Arecoline induced elevation of SSEA-4 expression in a dose-dependent manner
Li et al., 2009 [47]	Fibroblasts (FBs), fibroblasts with arecoline (FB20), fibroblasts co-cultured with keratinocytes (FB + KC0), and fibroblasts co-cultured with arecoline treated keratinocytes (FB + KC20)	Collagen concentration, MMP activity, TIMP concentration	MMP-9 secreted in a co-culture group and MMP2 activation elevated.<br><br>Collagen production increases in oral FBs induced by indirect arecoline stimulation and requires the interaction of both KCs with FBs.

			TIMP-1 increased in FB + KC20 group, playing an important role in collagen disorder of OSF.
Li et al., 2014 [39]	Keratinocytes of the naturally immortalised normal cell line (HaCaT) and the human normal fibroblast cell line (Hel) were challenged with arecoline	Cell viability, apoptosis, morphological changes, expression and activation of cleaved-Bid, cleaved-PARA, and cleaved-caspase-3	Arecoline induced morphological changes in HaCaT cells, suppressed their viability and promoted apoptosis in a dose-dependent manner<br><br>HaCaT apoptosis is associated with increased expression and activation of cleaved-Bid, cleaved-PARA, and cleaved-caspase-3
Tsai et al., 2003 [59]	Human buccal mucosa were challenged with arecoline.	COX-2	Arecoline induced elevation of COX-2 expression.
Tseng et al., 2014 [58]	EAHY cells derived from a human endothelial hybrid cell line were challenged with arecoline	Cell morphology, cytotoxicity, migration, attachment to U937 cells	Arecoline induced morphological changes to EAHY cells<br><br>Arecoline was cytotoxic to EAHY cells<br><br>Arecoline inhibited the migration of EAHY cells<br><br>Arecoline enhanced the adhesion of U937 cells to EAHY cells
Shieh et al., 2004 [67]	Human buccal mucosal fibroblasts	Collagen phagocytosis	Arecoline inhibited collagen phagocytosis

	were challenged with arecoline		by fibroblasts in a dose-dependent manner
Khan et al., 2015 [26]	Primary human gingival fibroblast (hGF) cells and human keratinocytes (HaCaT) were challenged with arecoline	Epithelial atrophy; mechanisms of cytotoxicity and proliferation.	Arecoline with copper enhances epithelial cytotoxicity and increases apoptosis suggested to lead to epithelial atrophy
Zheng et al., 2018 [34]	Normal primary human oral mucosal fibroblasts were challenged with arecoline	Epithelial-Mesenchymal Transition of hOMFs, p53 levels, LSD1 effects	Arecoline promoted EMT, resulted in hypermethylation of promoter TP53 with downregulation of p53 levels, and induced LSD1 which demethylated H3K27me1 and H3K4me2 resulting in p53 downregulation
Zhou et al., 2013 [40]	HaCaT epithelial and Hel fibroblast cell lines from OSF patients were challenged with arecoline	Effect on cell lines, cell morphology, cell proliferation and cell cycle arrest	Arecoline inhibits HaCaT epithelial cell proliferation, affects cell morphology, and induces cell cycle arrest in G1/S phase and subsequent survival in a dose-dependent manner. Arecoline treatment did not significantly alter Hel cell cycle distribution.
Zheng et al., 2015 [27]	HaCaT cells in OSF tissue compared to normal buccal mucosal tissue challenged with arecoline	EMT related gene expression: E-cadherin, N-cadherin, CK19, and vimentin and effect on miR-203, SFRP4 and TM4SF1	Arecoline decreased the expression of miR-203 and SFRP4 and increased TM4SF1 expression.<br><br>Arecoline enhances cell growth of HaCaT cells, attenuating cell proliferation.

			Arecoline affected the expression of EMT-related genes in a dose-dependent manner
Tsai et al., 2009 [74]	OSF fibroblasts and normal buccal mucosa fibroblasts challenged with arecoline	HO-1 expression	Arecoline induced elevation of HO-1 mRNA and protein expression in a dose dependent manner

Additional references (present in Supplementary Table S1 only)

[113] Wang C, Kadigamuwa C, Wu S, Gao Y, Chen W, Gu Y, Wang S, Li X. RNA N6-Methyladenosine (m6A) Methyltransferase-like 3 Facilitates Tumorigenesis and Cisplatin Resistance of Arecoline-Exposed Oral Carcinoma. Cells. 2022;11(22):3605.
[114] Yang HW, Yu CC, Hsieh PL, Liao YW, Chu PM, Yu CH, Fang CY. Arecoline enhances miR-21 to promote buccal mucosal fibroblasts activation. J Formos Med Assoc. 2021;120(4):1108-1113.
[115] Islam S, Uehara O, Matsuoka H, Kuramitsu Y, Adhikari BR, Hiraki D, Toraya S, Jayawardena A, Saito I, Muthumala M, Nagayasu H, Abiko Y, Chiba I. DNA hypermethylation of sirtuin 1 (SIRT1) caused by betel quid chewing-a possible predictive biomarker for malignant transformation. Clin Epigenetics. 2020;12(1):12.
[116] Chang MC, Ho YS, Lee PH, Chan CP, Lee JJ, Hahn LJ, Wang YJ, Jeng JH. Areca nut extract and arecoline induced the cell cycle arrest but not apoptosis of cultured oral KB epithelial cells: association of glutathione, reactive oxygen species and mitochondrial membrane potential. Carcinogenesis. 2001;22(9):1527-35.
[117] Jeng JH, Hahn LJ, Lin BR, Hsieh CC, Chan CP, Chang MC. Effects of areca nut, inflorescence piper betle extracts and arecoline on cytotoxicity, total and unscheduled DNA synthesis in cultured gingival keratinocytes. J Oral Pathol Med. 1999;28(2):64-71.
[118] Chang YC, Hu CC, Tseng TH, Tai KW, Lii CK, Chou MY. Synergistic effects of nicotine on arecoline-induced cytotoxicity in human buccal mucosal fibroblasts. J Oral Pathol Med. 2001;30(8):458-64.
[119] Liao YW, Yu CC, Hsieh PL, Chang YC. miR-200b ameliorates myofibroblast transdifferentiation in precancerous oral submucous fibrosis through targeting ZEB2. J Cell Mol Med. 2018;22(9):4130-4138.
[120] Yu CC, Liao YW, Hsieh PL, Chang YC. Targeting lncRNA H19/miR-29b/COL1A1 Axis Impedes Myofibroblast Activities of Precancerous Oral Submucous Fibrosis. Int J Mol Sci. 2021;22(4):2216.
[121] Yu CC, Yu CH, Chang YC. Aberrant SSEA-4 upregulation mediates myofibroblast activity to promote pre-cancerous oral submucous fibrosis. Sci Rep. 2016;6:37004.
